# Repeated measures of mammographic density and texture to evaluate prediction and risk of breast cancer: a systematic review of the methods used in the literature

**DOI:** 10.1007/s10552-023-01739-2

**Published:** 2023-06-20

**Authors:** Akila Anandarajah, Yongzhen Chen, Carolyn Stoll, Angela Hardi, Shu Jiang, Graham A. Colditz

**Affiliations:** 1 Division of Public Health Sciences, Department of Surgery, Washington University School of Medicine, 660 S Euclid Ave MSC 8100-0094-2200, Saint Louis, MO 63110 USA; 2 Saint Louis University School of Medicine, Saint Louis, MO USA; 3 Bernard Becker Medical Library, Washington University School of Medicine, MSC 8132-12-01, 660 S Euclid Ave, Saint Louis, MO 63110 USA

**Keywords:** Breast density, Mammography, Parenchymal patterns, Risk prediction, Texture

## Abstract

**Purpose:**

It may be important for women to have mammograms at different points in time to track changes in breast density, as fluctuations in breast density can affect breast cancer risk. This systematic review aimed to assess methods used to relate repeated mammographic images to breast cancer risk.

**Methods:**

The databases including Medline (Ovid) 1946-, Embase.com 1947-, CINAHL Plus 1937-, Scopus 1823-, Cochrane Library (including CENTRAL), and Clinicaltrials.gov were searched through October 2021. Eligibility criteria included published articles in English describing the relationship of change in mammographic features with risk of breast cancer. Risk of bias was assessed using the Quality in Prognostic Studies tool.

**Results:**

Twenty articles were included. The Breast Imaging Reporting and Data System and Cumulus were most commonly used for classifying mammographic density and automated assessment was used on more recent digital mammograms. Time between mammograms varied from 1 year to a median of 4.1, and only nine of the studies used more than two mammograms. Several studies showed that adding change of density or mammographic features improved model performance. Variation in risk of bias of studies was highest in prognostic factor measurement and study confounding.

**Conclusion:**

This review provided an updated overview and revealed research gaps in assessment of the use of texture features, risk prediction, and AUC. We provide recommendations for future studies using repeated measure methods for mammogram images to improve risk classification and risk prediction for women to tailor screening and prevention strategies to level of risk.

**Supplementary Information:**

The online version contains supplementary material available at 10.1007/s10552-023-01739-2.

## Introduction

Evolving technology from film mammograms to digital images has changed the sources of data and ease of access to study a range of summary measures from breast mammograms and risk of breast cancer [1]. In particular, given women have repeated mammograms as part of a regular screening program [2–4], and access to repeated digital images has become more feasible in real time for risk classification. Improved risk classification is fundamental to counseling women for their risk management [5, 6].

The leading measure for risk categorization extracted from mammograms is breast density [7, 8]. This is now widely used and reported with many states requiring return of mammographic breast density measures to women as part of routine screening. Mammographic breast density is a strong reproducible risk factor for breast cancer across different approaches used to measure it (clinical judgement or semi/automated estimation) [7]. Mammographic breast density has typically been measured as an average value across both left and right breasts to relate to risk of subsequent breast cancer. However, change in breast density has been much less frequently studied. The growing access to the large data from digital mammograms encourages a reassessment of the approaches employed to assess change in density and risk of subsequent breast cancer [9, 10].

Guidelines recommend screening mammography from age 45 (American Cancer Society [2]) or 50 (US Preventive Services Task Force [3]), with either annual or biennial mammography [4]. Women generally have a series of repeated mammograms (longitudinal data). Additionally, these recurring screening mammograms capture both the left and right breast. Despite the availability of bilateral longitudinal images, general decision making is still based on mammographic breast density at a point in time, averaged between the two breasts [11], to forecast the overall breast cancer risk. While a growing number of studies use more than just baseline mammogram values which could improve risk classification and is promising for clinical decision making, we note there is no systematic review and summary of these studies, although a recent publication reported results from 9 studies and combined the results to show a positive association between increase in the Breast Imaging Reporting and Data System (BI-RADS) density category and increase in breast cancer risk [12]. However, this publication only included association studies describing the magnitude of risk for change in density compared to those with no change. A richer summary of methods used to classify density and other features on mammograms and evaluate change in relation to risk can identify common approaches and help guide the use of change for breast cancer risk prediction. While texture features, such as calcification, masses, and anatomically oriented texture features, are important breast cancer risk predictors independent of mammographic breast density, their influence has been much less studied. Studies show that they can improve prediction model performance when added to breast density, and because they are usually machine-derived, this reduces bias in their identification [13–20]. Furthermore, a recent studies show improved performance over Tyrer Cuzick [21] and others show sustained performance with external validation of a mammography-based risk model [15]. Therefore, we undertook the current systematic review including prediction studies using change in mammographic breast density or other features for risk classification in addition to association studies.

We aim to summarize the methods used, the time from mammogram to diagnosis of breast cancer, methods for analysis of data from either one or both breasts (averaged or assessed individually), and identify gaps in evidence to prioritize future studies.

## Methods

### Eligibility criteria

#### Population

We considered all studies of adult women (at least 18 years old) involving primary data. Abstract-only papers, review articles, and conference papers were excluded.

#### Intervention

We included studies measuring change in mammographic features between mammograms. A study had to use at least two different mammograms to be included.

#### Outcomes

Our primary outcomes of interest were risk of breast cancer, including both invasive and in situ cancers, and differences in mammographic features over time. Presence of breast cancer was required to be dichotomized (yes/no), and analysis of other risks (e.g., risk of interval vs. screen-detected cancer) were not included. Studies were required to assess the relationship of the change in mammographic features with risk of breast cancer.

Only studies available in English were included.

### Information sources

The published literature was searched using strategies designed by a medical librarian (AH) for the concepts of breast density, mammography, and related synonyms. These strategies were created using a combination of controlled vocabulary terms and keywords and were executed in Medline (Ovid) 1946-, Embase.com 1947-, CINAHL Plus 1937-, Scopus 1823-, Cochrane Library (including CENTRAL), and Clinicaltrials.gov. Results were limited to English using database-supplied filters. Letters, comments, notes, and editorials were also excluded from the results using publication type filters and limits.

### Search strategy

An example search is provided below (for Embase).

('breast density'/exp OR ( (breast NEAR/3 densit*):ti,ab,kw OR (mammary NEAR/3 densit*):ti,ab,kw OR (mammographic NEAR/3 densit*):ti,ab,kw)) AND ('mammography'/deOR mammograph*:ti,ab,kwOR mammogram*:ti,ab,kwOR mastrography:ti,ab,kwOR ‘digital breast tomosynthesis’:ti,ab,kwOR ‘x-ray breast tomosynthesis’:ti,ab,kw)NOT ('editorial'/it OR 'letter'/it OR 'note'/it) AND [english]/lim.

The search was completed for the first time on September 9, 2020, and was run again on October 14, 2021 to retrieve citations that were published since the original search. The second search was date limited to 2020–October 14, 2021. Full search strategies are provided in Supplementary File 1.

### Selection process

Two reviewers (AA, CS) worked independently to review the titles and abstracts of the records. Next, the two reviewers independently screened the full-text of the articles that they did not reject and indicated those measuring mammographic features over time, which were ultimately eligible for inclusion. Any disagreements of which articles to include were resolved by consensus.

Reference lists of included studies were hand searched to find additional relevant studies.

### Data collection process

We created a data extraction sheet which two reviewers (AA, YC) used to independently extract data from the included studies. Disagreements were resolved by a third reviewer. If included studies were missing any desired information, any additional papers from the work cited, such as previous reports, methods papers, or protocols, were reviewed for this information.

### Data items

Any estimate of change in a mammographic feature over time or risk of breast cancer was eligible to be included. Predictive ability could be evaluated using an area under the curve, hazard ratio, odds ratio, or relative risk. Change could be reported as a percentage or an absolute value. No restrictions on follow-up time were placed. For studies that reported multiple risk estimates, we prioritized the primary models which were discussed in the results section of the paper. If all models were discussed equally, then we listed the models with the best ability to predict breast cancer. For studies that reported multiple types of change, we prioritized the primary types which were discussed in the results section of the paper. If all types were discussed equally in the results section of the paper, then we listed the most frequent types of change observed in women using data listed in the tables.

We collected data on:

- The report: author, publication year;
- The study: location/institution, number of cases, number of controls;
- The research design and features: lapsed time from mammogram to diagnosis;
- The mammogram: machine type, mammogram view (s), breast (s) used for analysis, time between mammograms, number of mammograms;
- The model: how density was measured, type of model, baseline variable (s), texture feature (s), prediction horizon.

### Risk of bias

The quality of the included studies was assessed using the Quality in Prognostic Studies (QUIPS) tool [22]. Risk of bias was rated as high, moderate, low, or unclear by two reviewers (AA and CS) across six domains including study participation, study attrition, prognostic factor measurement, outcome measurement, study confounding, and statistical analysis and reporting. Raters independently recorded supporting information and justification for judgements of risk of bias for each domain. Any disagreements were resolved by consensus.

For the prognostic factor measures domain, studies that used discrete categories of density were rated as high-risk of bias and those that used continuous measures assessed by machine were rated as low-risk of bias. For the study confounding domain, studies that adjusted for age, body mass index, menopausal status, and hormone therapy were considered to have a low-risk of bias.

### Registration and protocol

This review was not registered, and a protocol was not prepared.

## Results

**Fig. 1:**
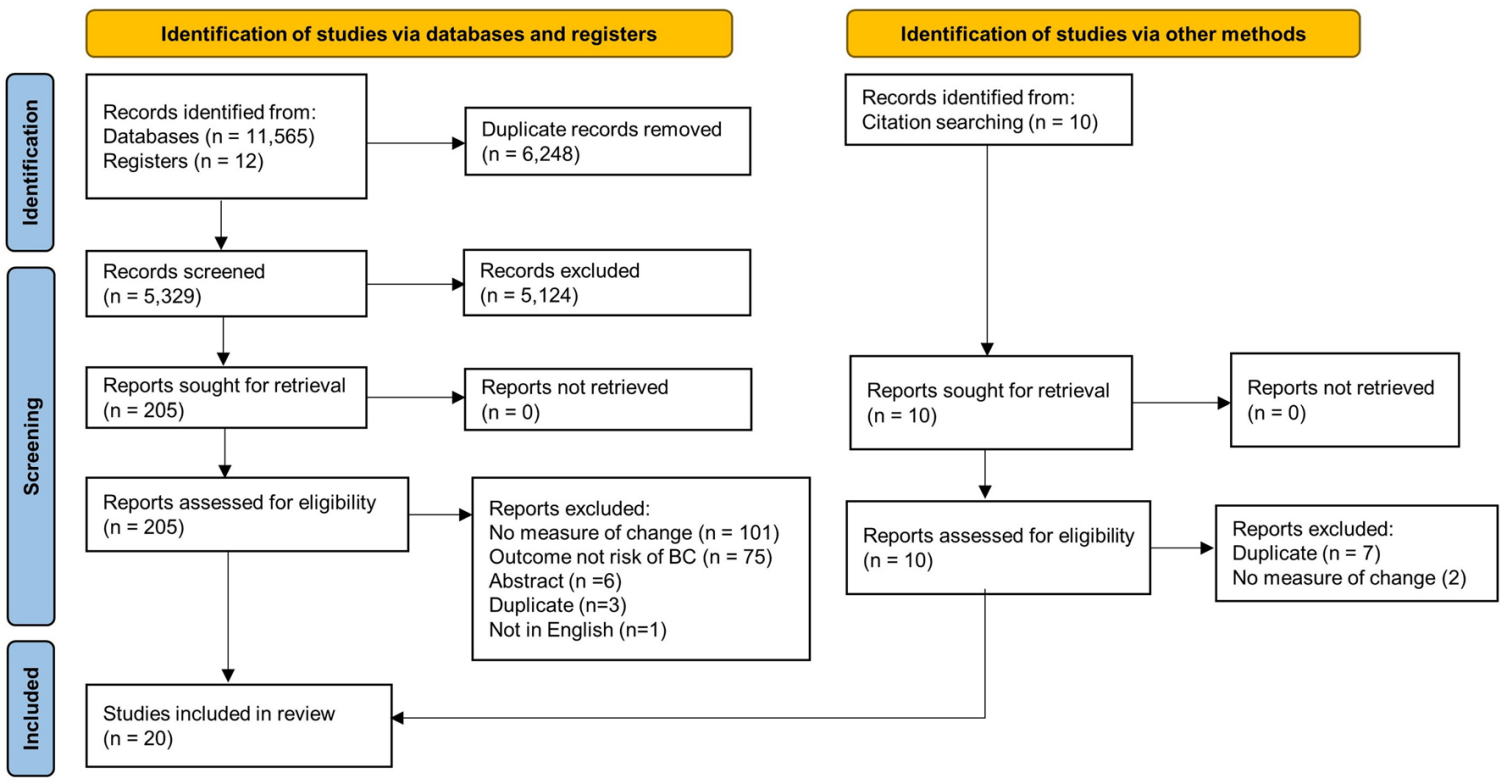
PRISMA 2020 flow diagram

The search and study selection process is shown in Fig. 1. A total of 11,111 results were retrieved from the initial database literature search and imported into Endnote. Eleven citations from ClinicalTrials.gov were retrieved and added to an Excel file library. After removing duplicates 4,863 unique citations remained for analysis. The search was run again in October 2021 to retrieve citations that were published since the original search. A total of 1,633 results were retrieved and imported to Endnote. After removing duplicates, including duplicates from the original search, 466 unique citations were added to the pool of results for analysis. Between the two searches a total of 11,577 results were retrieved, and there were 5,329 unique citations.

Of the 5,329 unique citations, 5,124 were excluded based on review of title and abstract. Two hundred five full-text reports were retrieved and assessed for eligibility by two readers. Of these, One hundred eighty five were excluded for reasons such as not measuring change, not having risk of breast cancer as an outcome, being an abstract or a duplicate paper, or not being published in English.

Ten potential reports were identified from hand searching of citations. All of these were reviewed by full-text, and nine were excluded for being duplicates or not having a measure of change in a mammographic feature.

After fully screening search results, twenty studies meeting eligibility criteria were included in the review [23–42]. These twenty studies used two or more mammograms to relate change in density or other features to risk of breast cancer and met eligibility criteria as set out in the selection flow chart. See the Preferred Reporting Items for Systematic Review and Meta-Analyses (PRISMA) flow chart (Fig. 1).

The key descriptive features of the 20 eligible studies are summarized in Table 1. These rely on mammography film records (ten studies) though studies published from 2016 onwards often use digital images. Measures of breast density used in these studies are summarized in Table 1. BI-RADS (six studies) [28–30, 32, 33, 36] and Cumulus (five studies) [25, 26, 34, 35, 42] were the most commonly used methods for density assessment. Automated assessments were used on digital mammograms. Studies differed in the view used to forecast the overall breast cancer risk, with some only using the craniocaudal (three studies) [26, 35, 40] or mediolateral oblique (four studies) [23, 25, 31, 34] while others considered both (seven studies) [24, 27, 28, 32, 36, 38, 39]. The number of cases included in each study ranged from a low of fourty five cases [28] to a high of 22,781 in a Korean cohort study as shown in Supplementary Table 1 [33].

**Table 1:** Features of studies using repeated assessment of mammographic features included in systematic review (sorted by year published)

Author	Year	Machine type	View used (CC/MLO/both)	Side used (left/right/both/avg)	Density (BI-RADS categories/continuous)
Salminen [37]	1998	Film	NR	Avg	Wolfe’s classification
van Gils [41]	1999	Film	Up until 1981/1982 the lateromedial view was used and, after that time, the MLO view	Ipsilateral	Fully computerized method
Maskarinec [35]	2006	Film	CC	Avg	Cumulus
Kerlikowske [30]	2007	NR	NR	Both	BI-RADS
Vachon [40]	2007	Film	CC	Both	Computer-assisted thresholding program
Lokate [34]	2013	Over 99% of the mammograms were film	MLO	Left	Cumulus
Work [42]	2014	Film	NR	Left	Cumulus
Kerlikowske [29]	2015	NR	NR	NR	BI-RADS
Busana [25]	2016	Film	MLO	Left for Cumulus. Left and avg for ImageJ	Cumulus and ImageJ-based method
Humphrey [27]	2016	Digital (not specified further)	Both	both	Volpara
Khoo [31]	2016	Film	MLO	Contralateral for cases, random side chosen for controls	New framework for fully automatically measuring breast density and detecting change in density
Tan [39]	2016	Digital (not specified further)	Both	Both	Computer-aided detection scheme
Byrne [26]	2017	Film	CC	Contralateral for cases, random side chosen for controls	Cumulus and Madena
Brandt [24]	2019	Hologic Selenia	Both	Both	Volpara
Román [36]	2019	Film and digital (not specified further)	Both	NR	BI-RADS
Azam [23]	2020	GE Medical Systems, Philips Healthcare, Sectra Imtec AB and FUJI	MLO	Contralateral for cases, random side chosen for controls	STRATUS
Kim [32]	2020	Hologic Selenia Dimensions and General Electric Senographe 2000D/DMR/DS A21	Both	Both	BI-RADS
Sartor [38]	2020	Siemens Novation and Inspiration and GE Senograph	Both	Both	Libra
Kang [28]	2021	GE Senograph DS/ESSENTIAL	Both	Both	BI-RADS
Kim [33]	2021	NR	NR	NR	BI-RADS

*avg* average, *BI-RADS* Breast Imaging Reporting and Data System, *CC* craniocaudal, *MLO* mediolateral oblique, *mmg* mammogram, *NR* not reported

**Fig. 2:**
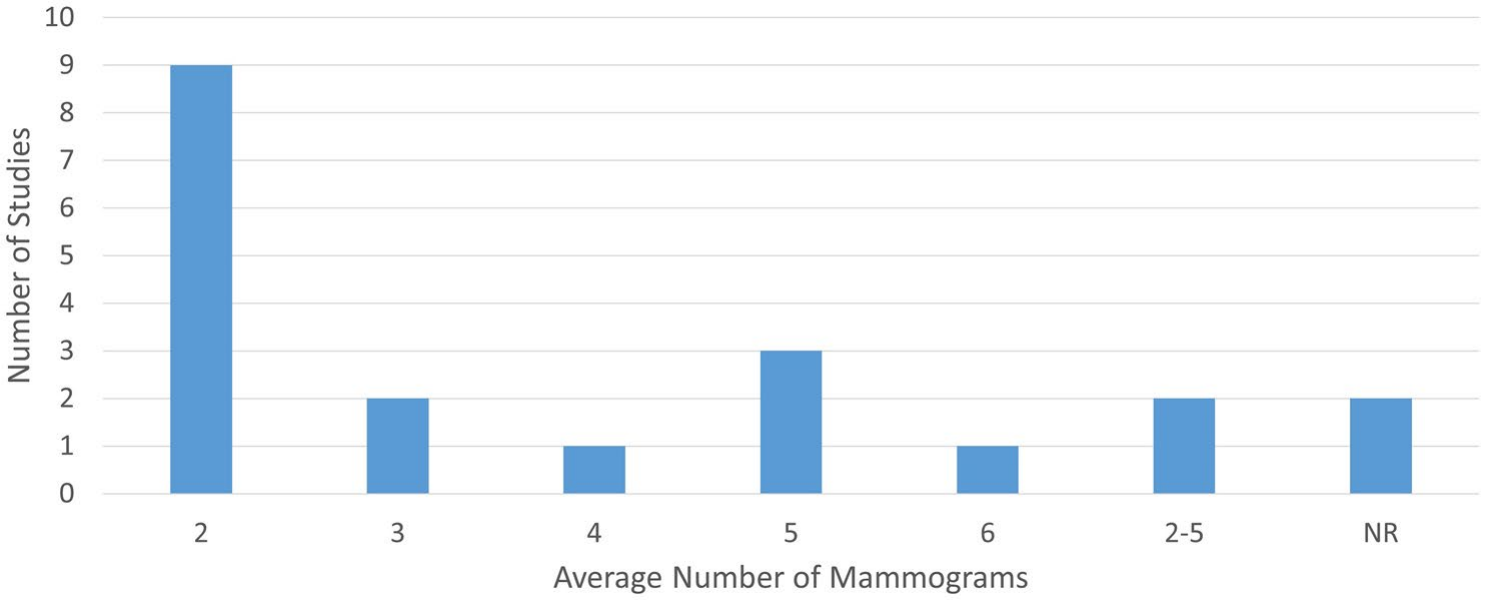
Number of mammograms per study

Figure 2 shows that from the twenty studies only nine used more than two images separated in time to assess change in relation to risk [23, 25, 34, 35, 37–41]. Supplementary Table 2 shows that the time between mammograms varied across studies from one to a median of 4.1 years reflecting differences in guidelines and screening practices across countries. Furthermore, these studies used varying statistical methods to model change and covariates were generally included, such as age, body mass index, and menopausal status. However, the covariates used to adjust estimates of association varied substantially across these studies.

Data from studies of change in mammographic density or other features and subsequent risk incorporated into models that are evaluated using an area under the curve (AUC) are summarized in Table 2 [24, 29, 31, 39, 40]. There is much variation in approaches to analysis used to relate change in breast density or other mammographic features to breast cancer risk. Approaches included change in BI-RADS category, change from first to last image (ignoring intermediate images), and change in density as a continuous measure.

**Table 2:** Models of repeated measures of mammographic features and incidence of breast cancer reporting AUC (sorted by average number of mammograms used)

Author	Year	AUC (baseline with density)	Overall AUC (with change in density or texture features)
Kerlikowske^a^	2015	5-year risk model: 0.635 (0.635 from fivefold cross-validation). 10-year risk model: 0.622. 5-year risk model among women who changed density categories: 0.630 (0.629 from fivefold cross-validation)	5-year risk model: 0.640 (0.639 from fivefold cross-validation). 10-year risk model: 0.628. 5-year risk model among women who changed density categories: 0.641 (0.639 from fivefold cross-validation)
Khoo	2016	0.566	0.590
Brandt	2019	0.52 for VPD, 0.53 for DV	0.54 for VPD, 0.56 for DV
Tan	2016	0.730 for prior 1, 0.710 for prior 2, 0.666 for prior 3	NR
Vachon	2007	NR	0.65

*AUC* area under curve, *NR* not reported

^a^Kerlikowske 2015 uses change in density to predict future risk of breast cancer, whereas other studies report association between change in mammographic characteristics and risk of breast cancer

Kerlikowske et al. showed modest improvement in estimating 5- and 10-year risk with AUC increasing from 0.635 using only one measure of density to 0.640 using two measures [29]. Brandt et al. showed similar modest change in AUC to discriminate cases from controls using volumetric percent density change in cancerous breast and normal breast from 0.52 to 0.54 after adding change in density to a model incorporating age, body mass index (BMI), change in BMI, and time between mammograms. However, the time horizon appears to be the time between the two mammograms used for this study (median time 3 years) [24]. Tan et al. on the other hand evaluated bilateral asymmetry of breast density between left and right breast as a marker of near-term cancer risk, comparing current mammograms to the three most recent which had been previously interpreted as negative [39].

In Supplementary Table 3, we summarize the number of mammograms used and the prediction horizon, which is how far ahead the model predicts risk of breast cancer. Only 1 study (Kerlikowske et al.) [29] based on change in BI-RADS category between two mammograms) reported a prediction horizon of 5 and 10 years. In others, the prediction horizon was not clearly defined with investigators using the next screening mammogram [24, 31, 39, 40, 43].

Other measures of association (relative risk, hazard ratio (HR), odds ratio (OR)) are reported in studies that focus on the association between mammographic features and cancer risk. The statistical methods used to model change and assumptions including the breast imaged (ipsilateral or contralateral to the cancer) and approach to comparing cases and controls for 15 other association studies are summarized in Supplementary Table 4 [23, 25–28, 30, 32–38, 40–42]. Some studies used change in BI-RADS category while others had continuous breast density generated from machine-derived measures and then categorized change from the variable.

Many of these studies also observed increased breast density over time was associated with an increase in the risk of breast cancer [23, 26, 28, 30, 32, 34, 36, 37, 41, 42] However, some results were not statistically significant [23, 26, 28, 32, 34, 37, 40, 42], often due to wide confidence intervals [23, 26, 28, 32, 37, 42] For example, van Gils et al. showed women whose mammographic density increased from 5–25 to > 25% had a significantly increased breast cancer risk (OR 6.9 95% CI 2.1–22.9) compared to those with a persisting density of 5–25% [41] On the other hand, Work et al. reported that a > 5% increase in percent density was positively associated with breast cancer (OR 2.55 95% CI 0.63–10.26) compared to women with *a* < 5% increase or decrease, but these results were non-significant [42].

Results from the assessment of risk of bias are shown in Supplementary Table 5. While many studies demonstrated similar risk of bias within specific domains, there is some variability, especially for prognostic factor measurement and study confounding. For prognostic factor measurement, studies that used categorical approaches such as BI-RADS or averaged the left and right breasts had a higher risk of bias than those using continuous measures assessed by machine approaches. For study confounding, studies that adjusted for age, body mass index, menopausal status, and hormone therapy were considered to have a lower risk of bias.

Study reporting impacted our ability to rate risk of bias. Only one study reported information to judge study attrition risk of bias [26], leaving most with an unclear risk of bias. Likewise, for study population, most studies did not report on the characteristics of the source population [23–28, 30–35, 37–42] making it difficult to determine whether the population of interest was adequately represented.

## Discussion

We identified twenty studies addressing change in breast density or other mammographic features and risk of breast cancer. Of these, nine had only two images [24, 26, 27, 29–31, 33, 36, 42] giving only modest ability to detect an association between change in density and risk of breast cancer. Only five studies report AUC for their analysis [24, 29, 31, 39, 40], and four of these use this measure to summarize discrimination of the cases from the controls [24, 31, 39, 40]. Only Kerlikowske et al. uses change in density categories from BI-RADS classification to predict 5- and 10-year risk. In that study, adding change in density to the prediction model gave a modest improvement in model performance [29]. While Kerlikowske et al. used AUC to determine whether change in mammographic features predicts risk of subsequent breast cancer, the other studies were association studies and report a measure of association between change and breast cancer. While these studies provide evidence of an association, they did not assess prediction performance of future risk, limiting their clinical translation [44]. Overall, approaches to analysis of repeated mammogram images reflect the underlying approach to density (categorical or continuous) and this variation further limits interpretation of this body of evidence.

Variation in risk of bias observed in these studies reflect the variation in methods particularly in prognostic factor measurement and consideration of confounding. Level of reporting impacted our ability to fully assess risk of bias in these studies.

It is difficult to draw conclusions about the differences in results and overall conclusions across the different measurements of change summarized here. Since 2016 analyses draw extensively on digital mammograms with a number of approaches to summarizing breast density and change in density. For example, only one study reported mammographic density change in terms of breast dense area [34]. In general, density decreased over time consistent with published literature [45]. We observed no substantial differences in the results or overall conclusions between studies that used different methods for measuring change in mammographic density, such as reporting absolute change using subtraction as compared to reporting relative change using the ratio of change from baseline. While studies still report change in BI-RADS categories [29, 30, 33, 36, 46, 47], continuous measures of density would be preferable.

Focus of these studies is predominantly on mammographic breast density with limited study of change in texture features. Only two studies look at change in texture features [31, 39]. A recent meta-analysis of change in density and breast cancer risk used data from four cohort studies and reported a pooled HR for increase in breast density compared to women with non-dense breast tissue (HR 1.61; 95% CI 1.33–1.92) for studies reporting hazard ratios and pooled OR for those reporting odds ratios (OR 1.98; 95% CI 1.31–3.0) [12] In that meta-analysis, decrease in breast density was associated with reduced risk compared to women with stable breast density (HR 0.78; 95% CI 0.71–0.87). Of note, a single study contributed multiple measures of change in density within this analysis without adjustment for use of a common reference group.

The twenty studies included in our review varied in the view of the breast used. A meta-analysis showed that the craniocaudal view may have a stronger association with breast cancer risk [48], but the inconsistent approaches to handling these views increases variability across studies. Interval between images used for change is also variable (range from as short as 1 year to median of 4.1 years). The majority of studies evaluate change in category of density; for example, BI-RADS is not a continuous measure of density. Not only can this lead to misclassification, but these categories are also subjective, varying between readers, while continuous measures generally involve automation.

With only two images used, change in category is limited and the shorter time interval between images reduces power to differentiate trajectories of mammographic features over time. There is a steady decline in mammographic breast density through midlife to menopause and beyond [45, 49]. This slow decrease over time makes a discrete change in category harder to capture and will be limited compared to use of continuous mammographic density measures that are now becoming more broadly available. Future studies using the continuous density measures may better capture change and the risk associated with these changes.

We might ask, given the low rate of decline in mammographic density with age as described [45, 49], is the interval used in studies sufficient to detect meaningful change? To address this gap in the literature, future studies should use repeated measures methods incorporating more mammographic images over longer time periods such as the recent paper by Jiang et al. [50].

Some studies indicate differences in mammographic density between the left and right breasts may be a risk factor for breast cancer, but more research is needed [14, 51, 52]. While breast cancer rarely develops simultaneously in both breasts, current models still utilize average mammographic density and/or other features between the two breasts in conducting the risk prediction. Although mammographic density from the two breasts appears to be highly correlated at the baseline, deviation between the two breasts may be better captured over time using repeated mammography. Based on this review of the literature, we conclude that longitudinal bivariate analysis [53] of mammograms which accounts for both breasts individually over long periods of time has been used in only one recently published breast cancer epidemiology study [50]. We note limited use of change measures for improving risk prediction. Reporting AUCs and net reclassification index to evaluate how much prediction has improved is critical. To address the issues present in these studies, we recommend that more studies assess the use of texture features with a focus on risk prediction and report AUC. Using continuous measures of density and more mammographic images over longer periods of time including the craniocaudal view may improve prediction of risk of subsequent breast cancer. Additionally, evaluating features in both breasts individually and accounting for confounding factors such as age, BMI, menopausal status, and hormone therapy use would strengthen the utility of models. Future studies should take care to thoroughly report methods used, especially for study attrition and study population, to allow for more accurate assessments of risk of bias.

It can be more difficult for radiologists to detect cancers in dense breasts, as the cancer can be masked by the dense tissue [8]. Because of this, mammography has reduced sensitivity in dense breasts. As a result, women with dense breasts may be at higher risk for interval cancers, which tend to be larger and more advanced than cancers that are detected on a mammogram, and they are associated with a lower survival rate [54]. Supplemental screening with breast magnetic resonance imaging (MRI) in women with dense breasts could potentially improve the detection of breast cancer. When high-risk screening MRI detects interval cancers, they are frequently cases of ductal carcinoma in situ (DCIS) and have a lower stage of primary tumor compared to cancers identified due to symptoms [55]. Additionally, MRI is more sensitive than mammography in women with dense breasts [56], and ultrasound may be an alternative adjunct to tomosynthesis [57].

There is increasing interest in bringing breast cancer risk reduction approaches to women according to their level of risk [5, 58]. In Canada, for example, ongoing research is evaluating the integration of risk prediction tools and single nucleotide polymorphisms into clinical preventive care to better balance benefits and harms of screening [59]. The WISDOM trial evaluates risk based screening [60]. Analyses also evaluate the potential of family history of breast cancer plus a polygenic risk score to guide screening before age 50 [61]. In Europe and the UK risk models are used to inform recommended screening and prevention [62]. These approaches require continuing translation of advances in biotechnology to focus treatment and prevention according to level of risk and refinement of the balance of risks and benefits of treatment or prevention [63]. Cancer prevention is often conceptualized as strategies that interrupt cancer pathways and maximize the short- and long-term benefits of prevention intervention [64–66] These strategies need to be applicable in real time in the clinical setting maximizing benefit-to-harm ratio [63, 67], such as in the context of increasing supplemental screening with breast MRI or ultrasound in women with dense breasts. Focus should also be placed on better use of repeated mammographic measures of breast features to stratify risk and identify both the high-risk groups and also the low-risk groups [68] to tailor screening and prevention strategies [59].

## Limitations

There are several limitations with the current review. Heterogeneity of the data did not allow for a meta-analysis. No joint models considering features through time and risk were also included in this review, as none were identified that met our inclusion criteria. This highlights a gap in methodology that future work could address. Additionally, systematic reviews are always subject to possible publication bias if all relevant studies have not been published. We used several strategies to reduce the risk of this including using a thorough search strategy designed by a medical librarian with expertise in searching for systematic reviews and searching clinicaltrials.gov for any ongoing studies.

## Conclusion

There exist several gaps in the methodology of studies assessing risk of breast cancer using change in mammographic features. Based on these, we provide recommendations for future studies to make use of accumulating image data. Despite current limitations in the literature, the more widespread use of digital mammography and availability of digital images repeated over time offers growing opportunities to improve risk classification and risk prediction for women.

### Supplementary Information

Below is the link to the electronic supplementary material.

Supplementary file1 (PDF 116 KB)

Supplementary file2 (PDF 353 KB)

## Data Availability

All searching strategy and all data for this manuscript is contained in the tables and supplementary materials that accompany the online version.
