# Supplementary material for: Repeated measures of mammographic density and texture to evaluate prediction and risk of breast cancer: a systematic review of the methods used in the literature
Source: Cancer Causes Control. 2023 Jun 20;34(11):939–48. doi: 10.1007/s10552-023-01739-2 (PMC10533570; doi:10.1007/s10552-023-01739-2)
Supplement: Supplementary file 1 — Supplementary file1 (PDF 116 KB) [file 10552_2023_1739_MOESM1_ESM.pdf]

Article title: Repeated measures of mammographic density and texture to evaluate prediction of breast cancer risk: a systematic review of the methods used in the literature
Journal name: Cancer Causes & Control
Author names: Akila Anandarajah, Yongzhen Chen, Carolyn Stoll, Angela Hardi, Shu Jiang, Graham A. Colditz
Affiliation and e-mail address of the corresponding author: Division of Public Health Sciences, Department of Surgery, Washington University School of Medicine, 660 S Euclid Ave MSC 8100-0094-2200, Saint Louis, MO 63110; colditzg@wustl.edu

Complete Search Strategies:
Search strategies designed and executed by Angela Hardi, MLIS

Embase.com
=3,919 results on 9/9/2020 (Limited to English; editorials, letters, and notes excluded from results)
Updated search (date limited to 2020-present): 602 on 10/14/2021
('breast density'/exp OR ((breast NEAR/3 densit*):ti,ab,kw OR (mammary NEAR/3 densit*):ti,ab,kw OR (mammographic NEAR/3 densit*):ti,ab,kw)) AND ('mammography'/de OR mammograph*:ti,ab,kw OR mammogram*:ti,ab,kw OR mastrography:ti,ab,kw OR 'digital breast tomosynthesis':ti,ab,kw OR 'x-ray breast tomosynthesis':ti,ab,kw) NOT ('editorial'/it OR 'letter'/it OR 'note'/it) AND [english]/lim

Ovid Medline All
= 2694 results on 9/9/2020 (Limited to English; editorials, comments, and letters excluded)
Updated search (date limited to 2020-present): 440 results on 10/14/2021
(Breast Density/ OR (breast adj3 densit*).ti,ab. OR (mammary adj3 densit*).ti,ab. OR (mammographic adj3 densit*).ti,ab.) AND (Mammography/ OR mammograph*.ti,ab. OR mammogram*.ti,ab. OR mastrography.ti,ab. OR "digital breast tomosynthesis".ti,ab. OR "x-ray breast tomosynthesis".ti,ab.) NOT (comment.pt. OR editorial.pt. OR letter.pt.)

CINAHL Plus
=978 results on 9/9/2020; (Limited to English and these publication types: Clinical Trial, Corrected Article, Journal Article, Meta Analysis, Meta Synthesis, Practice Guidelines, Proceedings, Protocol, Randomized Controlled Trial, Research, Review, Systematic Review)
Updated search (dated limited to 2020-present): 135 results on 10/14/2021
((MH "Breast Tissue Density") OR AB(breast N3 densit*) OR TI(breast N3 densit*) OR AB(mammary N3 densit*) TI(mammary N3 densit*) OR AB(mammographic N3 densit*) OR TI(mammographic N3 densit*)) AND ((MH "Mammography") OR AB(mammograph*) OR TI(mammograph*) OR AB(mammogram*) OR TI(mammogram*) OR AB(mastrography) OR TI(mastrography) OR AB(“digital breast tomosynthesis”) OR TI(“digital breast tomosynthesis”) OR AB(“x-ray breast tomosynthesis”) OR TI(“x-ray breast tomosynthesis”))

Scopus
=3,162 results on 9/9/2020 (Limited to English; editorials, notes, letters, and book chapters excluded from results)
Updated search (date limited to 2020-present): 423 results on 10/14/2021
TITLE-ABS ((breast W/3 densit*) OR (mammary W/3 densit*) OR (mammographic W/3 densit*)) AND TITLE-ABS (mammograph* OR mammogram* OR mastrography OR "digital breast tomosynthesis" OR "x-ray breast tomosynthesis") AND (EXCLUDE (DOCTYPE , "no") OR EXCLUDE (DOCTYPE , "ch") OR EXCLUDE (DOCTYPE , "le") OR EXCLUDE (DOCTYPE , "ed")) AND (LIMIT-TO (LANGUAGE , "English"))

Cochrane Library
=358 results on 9/9/2020 (1 Cochrane Protocol and 357 results from CENTRAL Trials)
Updated Search (date limited 2020-present in CENTRAL Trials) = 32 results on 10/14/2021

ID	Search
#1	MeSH descriptor: [Breast Density] explode all trees
#2	((breast NEAR/3 densit*) OR (mammary NEAR/3 densit*) OR (mammary NEAR/3 densit*) OR (mammographic NEAR/3 densit*)):ti,ab,kw
#3	#1 OR #2
#4	MeSH descriptor: [Mammography] explode all trees
#5	(mammograph* OR mammogram* OR mastrography OR “digital breast tomosynthesis” OR “xray breast tomosynthesis”):ti,ab,kw
#6	#4 OR #5
#7	#3 AND #6

ClinicalTrials.gov
= 11 results (searched the “Other terms” field) on 9/9/2020
Updated Search = 12 results on 10/14/2021 (1 new result, added to the Excel library)
(“breast density” OR “mammary density”) AND (mammograph* OR mammogram* OR mastrography OR “digital breast tomosynthesis” OR “x-ray breast tomosynthesis”)
