# Supplementary material for: Repeated measures of mammographic density and texture to evaluate prediction and risk of breast cancer: a systematic review of the methods used in the literature
Source: Cancer Causes Control. 2023 Jun 20;34(11):939–48. doi: 10.1007/s10552-023-01739-2 (PMC10533570; doi:10.1007/s10552-023-01739-2)
Supplement: Supplementary file 2 — Supplementary file2 (PDF 353 KB) [file 10552_2023_1739_MOESM2_ESM.pdf]

Article title: Repeated measures of mammographic density and texture to evaluate prediction of breast cancer risk: a systematic review of the methods used in the literature
Journal name: Cancer Causes & Control
Author names: Akila Anandarajah, Yongzhen Chen, Carolyn Stoll, Angela Hardi, Shu Jiang, Graham A. Colditz
Affiliation and e-mail address of the corresponding author: Division of Public Health Sciences, Department of Surgery, Washington University School of Medicine, 660 S Euclid Ave MSC 8100-0094-2200, Saint Louis, MO 63110; colditzg@wustl.edu

Supplementary Table 1 Sample size and time from mammogram to diagnosis of cancer in studies included (sorted by year published)

Author	Year	# cases	# controls	Time from mammogram to diagnosis of cancer
Salminen(37)	1998	68	4013	At least 6 months
van Gils(41)	1999	108	400	Prior to diagnosis. Diagnosed with cancer between 1985 and 1994. Screening from 1975-1994.
Maskarinec(35)	2006	607	667	The earliest mammogram was taken 6.3 ± 4.0 years before diagnosis
Kerlikowske(30)	2007	2639	299316	Within 12 months of the last examination
Vachon(40)	2007	372	713	The time interval between the initial mammogram and the diagnosis of cancer or exam date in controls was 7.0 ± 1.5 years on average (range, 2.1-10.4).
Lokate(34)	2013	533	1367	Mammograms taken until diagnosis
Work(42)	2014	85	85	Cancer diagnosis occurred a median of 1.5 years after the second mammogram (range, 6 months to 9.4 years)
Kerlikowske(29)	2015	13715	708939	Diagnosed during follow-up period (which has mean = 6.6 years, range = 1 day to 10 years). Women diagnosed the 3 months following their second examination were excluded.
Busana(25)	2016	313	452	Only images taken at least 1 year prior to diagnosis were included
Humphrey(27)	2016	170	510	Cases had an initial negative mammographic screen and another three years later when cancer was diagnosed.
Khoo(31)	2016	250	250	Mammogram before diagnosis used
Tan(39)	2016	159	176	The average elapsed time between the “current” and each of “prior” #1, #2 and #3 studies was 1.16±0.41, 2.30±0.55 and 3.44±0.72 years, respectively.
Byrne(26)	2017	174	733	NR
Brandt(24)	2019	1160	2360	Within 2 months and 1–5 years before diagnosis
Román(36)	2019	1592	115796	Diagnosed within two years of the last screening examination in the study period
Azam(23)	2020	563	43247	Diagnosed during follow-up period (which has average = 5.4 years)
Kim(32)	2020	803	73446	Diagnosed during follow-up period (which has median = 6.1 years, interquartile range = 4.1-8.8 years, maximum = 13 years)
Sartor(38)	2020	51	102	The last mammogram was defined as the last screening round before diagnosis (76 %) or the diagnostic mammographic examination (24%)
Kang(28)	2021	45	3552	Diagnosed during follow-up period (which has median = 4.8 years, interquartile range 2.8–7.5 years)
Kim(33)	2021	22781	3278498	Excluded women diagnosed with breast cancer within 90 days after the second screening. Screening in both 2009 to 2010 and 2011 to 2012 with breast cancer incidence identified up to December 2017.

Abbreviations: NR = not reported

Supplementary Table 2 Time between mammograms, baseline variables, and texture features in studies included (sorted by increasing average number of mammograms used)

**Author**	**Year**	**Avg[a] number of mmgs used**	**Avg[a] time between mmgs**	**Avg[a] time from first to last mmg**	**Baseline variables**	**Non-density texture considered Y/N (if yes, what)**
Kerlikowske	2007	2	Median = 3.2 years	Median = 3.2 years	adjusted for mammography registry, time between the two screening examinations, age	N
Work	2014	2	Median = 4.0 years for cases (range 1-15) and 4.0 years for controls (range 1-14)	Median = 4.0 years for cases (range 1-15) and 4.0 years for controls (range 1-14)	Adjusted for age at first mammogram (Model 1), as well as parity, family history, menopausal status (Model 2) and additionally adjusted for baseline density (Model 3). Change in percent density is not adjusted.	N
Kerlikowske	2015	2	1.8 years	1.8 years	none	N
Humphrey	2016	2	3 years	3 years	adjusted for relevant density measure at initial screen	N
Khoo	2016	2	at most 3 years	at most 3 years	none	Y. Includes first 13 Haralick texture features computed for three scales and brightness.
Byrne	2017	2	1 year	1 year	adjusted for baseline BMI, age, clinical center, age at first birth, and parity	N
Brandt	2019	2	Median = 3.0 years for cases, 3.1 years for controls	Median = 3.0 years for cases, 3.1 years for controls	age, BMI, change in BMI, time between mammograms	N
Román	2019	2	Average = 5.8 years, median = 4.1 years	Average = 5.8 years, median = 4.1 years	adjusted for time between screening mammography examinations (offset), screening center, mammography type, and year of screen	N
Kim	2021	2	screened twice during 2009 to 2010 and 2011 to 2012	screened twice during 2009 to 2010 and 2011 to 2012	adjusted for age, age at menarche, menopausal status, age at menopause, number of children, breastfeeding duration, hormone replacement therapy among menopausal women, oral contraceptive use, family history in first-degree relatives, body-mass index, smoking status, drinking status during the past year and physical activity per week	N
Kim	2020	NR	women participated in an annual or biannual health examinations	NR	age adjusted only and adjusted for age, center, year of screening examination, smoking status, regular exercise, alcohol intake, educational level, BMI, history of diabetes, history of hypertension, history of cardiovascular	N

Author	Year	Avg[a] number of mmgs used	Avg[a] time between mmgs	Avg[a] time from first to last mmg	Baseline variables	Non-density texture considered Y/N (if yes, what)
					disease, family history of BC, female hormone medication use, and menopausal status	
Kang	2021	NR	Measured both pre and postmenopause. Excluded participants who had more than a 5-year interval between exams pre and postmenopause.	NR	Model 1 was adjusted for age and BMI. Model 2 was further adjusted for smoking status, alcohol consumption, family history of BC, diabetes, hypertension and dyslipidemia. Logistic regression model was adjusted for age at menopause and BMI.	N
Salminen	1998	2-5	2 years	NR	adjusted for age only and age, BMI, number of pregnancies, size of the breast	N
Azam	2020	2-5	18-24 months	Mean = 5.4 years	adjusted for age, BMI, and MD area at baseline	N
Maskarinec	2006	2.8	If available, mammograms for every second or third year were scanned	4.2 years for controls and 5.1 for cases	Predictor variables in the final model included age, the square of age, case status, ethnicity, BMI, soy intake early in life, soy intake as an adult, parity, age at menarche, age at first live birth, menopausal status, and HT use in the year of the mammogram.	N
Sartor	2020	3	Median = 4.5 years for cases and 4.7 years for controls	NR	none	N
Tan	2016	4	Elapsed time between the "current" and each of "prior" #1, #2 and #3 studies was 1.16, 2.30 and 3.44 years, respectively	Elapsed time between the "current" and each of "prior" #1, #2 and #3 studies was 1.16, 2.30 and 3.44 years, respectively	none	Y. Selected relevant features from 158 initial mammographic density, structural similarity, and texture based image features.
van Gils	1999	5	2 years	NR	adjusted for family history of BC, age at first birth/nulliparity, age at menarche, menopausal status and Quetelet index at the beginning of the study period	N
Lokate	2013	5	NR	Mean = 9.5 years	Linear mixed effects model was adjusted for BMI, height, parity, age at first delivery, number of children, menopausal status, contraceptive pill use, and hormone therapy use. All logistic regression analyses were adjusted for the same potential confounders as the linear mixed	N

Author	Year	Avg[a] number of mmgs used	Avg[a] time between mmgs	Avg[a] time from first to last mmg	Baseline variables	Non-density texture considered Y/N (if yes, what)
					effects model, plus the follow-up time between the first and last mammograms.	
Vachon	2007	For cases, 5.0. For controls, 5.2.	NR	Mean = for cases, 7.1 years. For controls, 7.0.	adjusted for BMI, menopausal status, BC in a first degree relative, age at first birth, number of births, and HT status	N
Busana	2016	For cases, 5. For controls, 7.	1 year	NR	adjusted for age, BMI, parity, and family history of BC	N

Abbreviations: avg = average, BC = breast cancer, BMI = body mass index, HT = hormone therapy, MD = mammographic density, mmg = mammogram, N = no, NR = not reported, Y = yes

[a] Average specified as mean or median when mentioned by study

Supplementary Table 3 Prediction horizon and change features of studies reporting AUC (sorted by average number of mammograms used)

**Author**	**Year**	**Avg[a] number of mmgs used**	**Prediction horizon (5/10 yr)**	**Change in density or texture features for cases**	**Change in density or texture features for controls**
Kerlikowske[b]	2015	2	5 and 10 year	A total of 63.5% of combined cases and controls had the same BI-RADS density on two sequential examinations while 17.9% had an increase in breast density category and 18.6% had a decrease. The most common combinations of changing density categories were heterogeneously dense on the earlier examination and scattered fibroglandular on the most recent examination (10.0%) and scattered fibroglandular densities on the earlier examination and heterogeneously dense on the most recent examination (9.9%).	A total of 63.5% of combined cases and controls women had the same BI-RADS density on two sequential examinations while 17.9% had an increase in breast density category and 18.6% had a decrease. The most common combinations of changing density categories were heterogeneously dense on the earlier examination and scattered fibroglandular on the most recent examination (10.0%) and scattered fibroglandular densities on the earlier examination and heterogeneously dense on the most recent examination (9.9%).
Khoo	2016	2	NR	NR	NR
Brandt	2019	2	NR	The cancerous (ipsilateral) breast VPD decreased 0.26% and the contralateral breast VPD decreased 0.39% for a difference of 0.13%. For DV, the ipsilateral breast decreased 2.10 cm^3 and the contralateral breast decreased 2.74 cm^3 for a difference of 0.63 cm^3.	The ipsilateral breast VPD decreased 0.29% and the contralateral breast VPD decreased 0.28% for a difference of -0.02%. For DV, the ipsilateral breast decreased 1.82 cm^3 and the contralateral breast decreased 1.89 cm^3 for a difference of 0.05 cm^3.
Tan	2016	4	NR	NR	NR
Vachon	2007	Mean for cases, 5.0. For controls, 5.2.	NR	Difference in percent density from earliest to latest mammogram: for the ipsilateral side, mean (SE) = -1.3% (7.5). For the contralateral side, mean (SE) = -1.5% (7.4). The difference in PD in the contralateral breast between cases and controls was 5.5% at 9 years before the cancer, 5.3%, at 5 years, and only 4.0% at 3 years. The corresponding values for the ipsilateral side were 4.9%, 4.9%, and 4.1%, respectively.	Difference in percent density from earliest to latest mammogram: for the ipsilateral side, mean (SE) = -1.2% (6.3). For the contralateral side, mean (SE) = -1.1% (6.5). The difference in PD in the contralateral breast between cases and controls was 5.5% at 9 years before the cancer, 5.3%, at 5 years, and only 4.0% at 3 years. The corresponding values for the ipsilateral side were 4.9%, 4.9%, and 4.1%, respectively.

Abbreviations: AUC = area under curve, avg = average, DV = dense volume, mmg = mammogram, NR = not reported, SE = standard error, VPD = volumetric percent density

[a] Average specified as mean or median when mentioned by study

[b] Kerlikowske 2015 uses change in density to predict future risk of breast cancer, whereas other studies report association between change in mammographic characteristics and risk of breast cancer

Supplementary Table 4 Analytical models used for repeated measures of mammographic features that do not report AUC (sorted by average number of mammograms used)

Author	Year	Avg[a] number of mmgs used	Prediction horizon (5/10 yr)	Risk other than AUC	Change in density or texture features for cases	Change in density or texture features for controls
Kerlikowske	2007	2	NR	OR of BC if BI-RADS breast density category increased from 1 to 2 (= 1.9, 95% CI = 1.4 to 2.6) and 1 to 3 (= 3.4, 95% CI = 2.0 to 5.7)	A total of 19.6% of all women had an increase in breast density category and 18.5% had a decrease. The majority of women had a BI-RADS breast density category of 2 or 3 on the first and last examination: 29.4% of women with BC had BI-RADS scores of 2 on the first and last screens, and 24.3% women with BC had scores of 3 on both screens.	A total of 19.6% of all women had an increase in breast density category and 18.5% had a decrease. The majority of women had a BI-RADS breast density category of 2 or 3 on the first and last examination: 30.0% of women without BC had BI-RADS scores of 2 on the first and last screens, and 22.9% of women without BC had scores of 3 on both screens.
Work	2014	2	NR	A >5% decrease in percent density was inversely associated with BC (OR=0.56, 95% CI = 0.15 to 2.17 for fully adjusted model), while a >5% increase in percent density was positively associated with BC (OR=2.55, 95% CI = 0.63 to 10.26); however, these associations were not statistically significant.	Percent density = +0.29% per year. Mean change = -2.67% (range of -34–16).	Percent density = -1.62% per year. Mean change = -5.35% (range of -48–49).
Humphrey	2016	2	NR	NR	PD change for affected breast = -0.4%, PD change for unaffected breast = -0.3%	PD change = -0.4% and -0.5%
Byrne	2017	2	NR	Controlling for baseline mammographic density, a 1% change in mammographic density increased BC risk 4%, but not statistically significantly, in women assigned placebos (OR = 1.04, 95% CI = 0.98 to 1.11). The increase in BC risk was not statistically significant (OR = 1.20, 95% CI = 0.40 to 2.97) comparing the highest to the lowest quintile of mammographic density change.	For combined cases and controls assigned placebo, mean mammographic density change = -0.05% with median of 0.0%	For combined cases and controls assigned placebo, mean mammographic density change = -0.05% with median of 0.0%
Román	2019	2	NR	Women whose density category	Most frequently, women	Most frequently, women remained at

Author	Year	Avg[a] number of mmgs used	Prediction horizon (5/10 yr)	Risk other than AUC	Change in density or texture features for cases	Change in density or texture features for controls
				increased from B to C or B to D had a RR of 1.55 (95% CI = 1.24 to 1.94) and 2.32 (95% CI = 1.48 to 3.63), respectively. The RR for women whose density increased from C to D was 1.51 (95% CI = 1.03 to 2.22).	remained at density category B at earliest and latest examination (33.1%). The proportion of women that remained at BI-RADS density C or D was greater for women with BC. 34.0% experienced a decrease and 12.5% experienced an increase in breast density category.	density category B at earliest and latest examination (40.8%). The proportion of women that remained at BI-RADS density A or B was significantly greater for women without BC. 25.8% had a decrease, and 11.8% had an increase in breast density category.
Kim	2021	2	5 year	For women with BI-RADS Category 4 during both screenings, the 5-year risk was 1.24% (95% CI = 1.19 to 1.28).	23.0% of combined cases and controls had a higher density category, and 22.2% had a lower density category in the second screening compared to the first screening.	23.0% of combined cases and controls had a higher density category, and 22.2% had a lower density category in the second screening compared to the first screening.
Kim	2020	NR	NR	Multivariable-adjusted HRs for incident BC comparing the regressed, developed, and persistent breast density groups with the "none" group were 1.81 (95% CI = 0.98 to 3.36), 1.47 (95% CI = 0.76 to 2.84), and 3.01 (95% CI = 1.94 to 4.69), respectively.	NR	NR
Kang	2021	NR	NR	In comparison to consistently nondense group, HR for consistently dense group = 2.31 (95% CI = 1.12 to 4.75) for fully adjusted model, HR for density decrease = 0.83 (95% CI = 0.19 to 3.60), and HR for density increase = 1.04 (95% CI = 0.24 to 4.42). In addition, compared to the participants with decreased breast density after menopause, participants with increased breast density had a four-fold greater risk of BC (HR = 4.27, 95% CI = 0.45 to 40.29).	For combined cases and controls, 199 (5.5%) experienced a density decrease and 185 (5.1%) a density increase pre to postmenopause. The other 89.4% of participants exhibited no changes in density; 641 (17.8%) had consistently dense breast tissue and 2,572 (71.5%) never exhibited dense breast.	For combined cases and controls, 199 (5.5%) experienced a density decrease and 185 (5.1%) a density increase pre to postmenopause. The other 89.4% of participants exhibited no changes in density; 641 (17.8%) had consistently dense breast tissue and 2,572 (71.5%) never exhibited dense breast.
Salminen	1998	2-5	NR	The age-adjusted RR of BC among women with unfavourable	For combined cases and controls, at the first screening	For combined cases and controls, at the first screening round, the prevalence of

Author	Year	Avg[a] number of mmgs used	Prediction horizon (5/10 yr)	Risk other than AUC	Change in density or texture features for cases	Change in density or texture features for controls
				parenchymal patterns of the breast at the first round were only marginally increased (RR varied from 1.5 to 1.3 among women with P2 to DY patterns). After taking into account the mammographic parenchymal pattern sequentially at the rounds preceding the diagnosis, the RRS varied from 2.6 to 4.7 and were statistically significant. There was only a small and not statistically significant increase in the risk of BC among those women whose breast patterns changed either from favorable to unfavorable (RR = 1.3, 95% CI = 0.2 to 9.8) or from unfavorable to favorable (RR = 1.2, 95% CI = 0.5 to 2.8) compared with women whose patterns remained favorable.	round, the prevalence of normal mammographic parenchymal pattern (N1) was 13% and the prevalence of DY pattern was about 4%. There was a drift in the mammographic parenchymal patterns from the unfavourable P2,DY types to the favourable N1,P1 types between the first and the last screening rounds. At the last screening round the prevalence of N1 patterns was 46% and that of DY patterns under 1%.	normal mammographic parenchymal pattern (N1) was 13% and the prevalence of DY pattern was about 4%. There was a drift in the mammographic parenchymal patterns from the unfavourable P2,DY types to the favourable N1,P1 types between the first and the last screening rounds. At the last screening round the prevalence of N1 patterns was 46% and that of DY patterns under 1%.
Azam	2020	2-5	NR	Compared with women with a decreased MD over time, no statistically significant difference in BC risk was seen for women with either stable MD or increasing MD (HR = 1.01, 95% CI = 0.82 to 1.23 and 0.98, 95% CI = 0.80 to 1.22, respectively). Among premenopausal women, there was a weak but statistically nonsignificant association between annual increase in MD greater than 10% and risk of BC (HR = 1.12, 95% CI = 0.77 to 1.64) compared with premenopausal women with an annual decrease in MD greater than 10%. Women ages 40-49 with an increase in annual MD greater than 10% had a	NR	NR

Author	Year	Avg[a] number of mmgs used	Prediction horizon (5/10 yr)	Risk other than AUC	Change in density or texture features for cases	Change in density or texture features for controls
				statistically nonsignificant 30% higher risk compared to perimenopausal women with greater than 10% annual MD reduction.		
Maskarinec	2006	mean = 2.8	NR	NR	For combined cases and controls, unadjusted percent densities differed by ~20% between age 40 and 60. We estimated the age-related decline as 5.63% per 10 years. The nonlinear effect of 1.64% per 10 years in the full model described the faster decline of densities over time earlier in life than later. The mean size of the total breast area was 25% larger at age 75 to 80 than at age 40 to 45, whereas the size of the dense areas decreased by 34% with age.	For combined cases and controls, unadjusted percent densities differed by ~20% between age 40 and 60. We estimated the age-related decline as 5.63% per 10 years. The nonlinear effect of 1.64% per 10 years in the full model described the faster decline of densities over time earlier in life than later. The mean size of the total breast area was 25% larger at age 75 to 80 than at age 40 to 45, whereas the size of the dense areas decreased by 34% with age.
Sartor	2020	3	NR	NR	Density change = -0.3%	Density change = +1.7%
van Gils	1999	5	10 year	In women with 5–25% density initially, we observed a trend of decreasing risk with diminishing density: when women with <5% density throughout the whole period formed the reference category, the OR for those who decreased from 5–25% to <5% density was 1.9 (95% CI = 0.6 to 6.1) in contrast to the OR of 5.7 (95% CI = 2.2 to 15.2) for those with persisting 5–25% density. The risk in women whose density pattern increased from 5–25% density to >25% density was slightly higher (OR = 6.9; 95% CI = 2.1 to 22.9) than that in women with persisting 5–25% density.	Majority of cases stayed at 5-25% density	Majority of controls stayed at 5-25% density
Lokate	2013	5	NR	For each percentage point	The mean decline in percent	The mean decline in percent density

Author	Year	Avg[a] number of mmgs used	Prediction horizon (5/10 yr)	Risk other than AUC	Change in density or texture features for cases	Change in density or texture features for controls
				decrease in mammographic density, OR = 1.01 (95% CI = 0.99 to 1.02). Those who increased in density by one or more categories seemed to have a slightly increased risk (statistically significant only for those increasing from the first to the second quartile of dense area, OR = 2.8, 95% CI = 1.3 to 6.1).	density between the first and last available mammograms was 10.8% for both BC cases and controls. The change in absolute dense area was not different for BC cases and controls (mean = -15.2 cm^2). The mean change in absolute nondense area was +12.3 cm^2. 47% of all women showed a decline; 49% stayed in the same category; and 4% showed an increase in percent density over an average period of 10 years. Generally, women in whom breast density decreased with one or more categories had a slightly lower risk of BC than did those who stayed in the same category, although not statistically significant.	between the first and last available mammograms was 10.8% for both BC cases and controls. The change in absolute dense area was not different for BC cases and controls (mean = -15.2 cm^2). The mean change in absolute nondense area was +7.3 cm^2. 47% of all women showed a decline; 49% stayed in the same category; and 4% showed an increase in percent density over an average period of 10 years. Generally, women in whom breast density decreased with one or more categories had a slightly lower risk of BC than did those who stayed in the same category, although not statistically significant.
Vachon	2007	Mean for cases, 5.0. For controls, 5.2.	NR	For contralateral PD, the ORs range from 1.0043 (for change in PD of -10 or quartile 1) to 0.9972 (for change in PD of +6.5 or quartile 4) and the confidence intervals exclude all values smaller than 0.99 and >1.02. These results were similar for the ipsilateral side [0.9997 (for change in PD of -10 or quartile 1) to 1.0002 (for change in PD of +6.5 or quartile 4)].	Difference in percent density from earliest to latest mammogram: for the ipsilateral side, mean (SE) = -1.3% (7.5). For the contralateral side, mean (SE) = -1.5% (7.4). The difference in PD in the contralateral breast between cases and controls was 5.5% at 9 years before the cancer, 5.3%, at 5 years, and only 4.0% at 3 years. The corresponding values for the ipsilateral side were 4.9%, 4.9%, and 4.1%, respectively.	Difference in percent density from earliest to latest mammogram: for the ipsilateral side, mean (SE) = -1.2% (6.3). For the contralateral side, mean (SE) = -1.1% (6.5). The difference in PD in the contralateral breast between cases and controls was 5.5% at 9 years before the cancer, 5.3%, at 5 years, and only 4.0% at 3 years. The corresponding values for the ipsilateral side were 4.9%, 4.9%, and 4.1%, respectively.
Busana	2016	For cases, 5. For controls, 7.	NR	Women with a high PD at baseline, which remained high over time, had the highest odds of developing BC relative to a woman with mean random	NR	Cumulus = -1.17% a year, Image-J = -1.07% a year. The linear component of the yearly rate of change in PD was more than twice as fast after the menopausal transition than prior to it for Cumulus (-

Author	Year	Avg[a] number of mmgs used	Prediction horizon (5/10 yr)	Risk other than AUC	Change in density or texture features for cases	Change in density or texture features for controls
				intercept and mean slope (OR: 8.10, 95% CI = 3.96 to 16.6 for Cumulus (left MLO) and 3.42, 95% CI = 2.00 to 5.48 for the ImageJ-based method (left–right MLO mean). In contrast, women with the lowest PD at baseline, despite a slight increase in their PD over time, had the lowest odds of developing BC (OR: 0.07, 95% CI = 0.03 to 0.16 for Cumulus (left MLO) and 0.23, 95% CI = 0.12 to 0.43 for the ImageJ-based method (left–right MLO mean).		1.10 % vs. -0.50 %, respectively). Similarly, the yearly rate coefficient in the ImageJ-based model was nearly twice as fast after the menopause (-1.16% vs. 0.67%, respectively).

Abbreviations: AUC = area under curve, avg = average, BC = breast cancer, BI-RADS = Breast Imaging Reporting and Data System, HR = hazard ratio, MD = mammographic density, MLO = mediolateral oblique, mmg = mammogram, NR = not reported, PD = percent density, OR = odds ratio, RR = relative risk, SE = standard error, yr = year

[a] Average specified as mean or median when mentioned by study

Supplementary Table 5 Risk of bias assessments for included studies using QUIPS (sorted by year published)

Author	Year	Study Participation	Study Attrition	Prognostic Factor Measurement	Outcome Measurement	Study Confounding	Statistical Analysis and Reporting
Salminen	1998	Low risk of bias	Unclear risk of bias	High risk of bias	Low risk of bias	Moderate risk of bias	Moderate risk of bias
van Gils	1999	Low risk of bias	Unclear risk of bias	Low risk of bias	Low risk of bias	Moderate risk of bias	Moderate risk of bias
Maskarinec	2006	Low risk of bias	Unclear risk of bias	Moderate risk of bias	Low risk of bias	Low risk of bias	Moderate risk of bias
Kerlikowske	2007	Low risk of bias	Unclear risk of bias	Moderate risk of bias	Low risk of bias	Moderate risk of bias	Moderate risk of bias
Vachon	2007	Low risk of bias	Unclear risk of bias	Low risk of bias	Low risk of bias	Moderate risk of bias	Moderate risk of bias
Lokate	2013	Low risk of bias	Unclear risk of bias	Low risk of bias	Low risk of bias	Moderate risk of bias	Moderate risk of bias
Work	2014	Low risk of bias	Unclear risk of bias	Low risk of bias	Low risk of bias	Moderate risk of bias	Moderate risk of bias
Kerlikowske	2015	Low risk of bias	Unclear risk of bias	Moderate risk of bias	Low risk of bias	Moderate risk of bias	Low risk of bias
Busana	2016	Low risk of bias	Unclear risk of bias	Moderate risk of bias	Low risk of bias	Moderate risk of bias	Moderate risk of bias
Humphrey	2016	Low risk of bias	Unclear risk of bias	Low risk of bias	Low risk of bias	Low risk of bias	Moderate risk of bias
Khoo	2016	Moderate risk of bias	Unclear risk of bias	Low risk of bias	Low risk of bias	High risk of bias	Moderate risk of bias
Tan	2016	Moderate risk of bias	Unclear risk of bias	Low risk of bias	Low risk of bias	High risk of bias	Moderate risk of bias
Byrne	2017	Low risk of bias	Moderate risk of bias	Low risk of bias	Low risk of bias	Low risk of bias	Moderate risk of bias
Brandt	2019	Low risk of bias	Unclear risk of bias	Low risk of bias	Low risk of bias	Moderate risk of bias	Moderate risk of bias
Román	2019	Low risk of bias	Unclear risk of bias	Moderate risk of bias	Low risk of bias	Moderate risk of bias	Moderate risk of bias
Azam	2020	Low risk of bias	Unclear risk of bias	Low risk of bias	Low risk of bias	Moderate risk of bias	Moderate risk of bias
Kim	2020	Low risk of bias	Unclear risk of bias	Moderate risk of bias	Moderate risk of bias	Low risk of bias	Moderate risk of bias
Sartor	2020	Low risk of bias	Unclear risk of bias	Low risk of bias	Low risk of bias	Moderate risk of bias	Moderate risk of bias
Kang	2021	Low risk of bias	Unclear risk of bias	Moderate risk of bias	Low risk of bias	Moderate risk of bias	Moderate risk of bias
Kim	2021	Low risk of bias	Unclear risk of bias	Moderate risk of bias	Low risk of bias	Low risk of bias	Moderate risk of bias

QUIPS = Quality in Prognostic Studies
